# Development and Effectiveness of a Mobile Health Intervention in Improving Health Literacy and Self-management of Patients With Multimorbidity and Heart Failure: Protocol for a Randomized Controlled Trial

**DOI:** 10.2196/35945

**Published:** 2022-04-29

**Authors:** Pilar Bas-Sarmiento, Martina Fernández-Gutiérrez, Miriam Poza-Méndez, Antonio Jesús Marín-Paz, Olga Paloma-Castro, José Manuel Romero-Sánchez, ASyAG_PPIC Team

**Affiliations:** 1 Institute of Research and Innovation in Biomedical Sciences of the Province of Cádiz (INiBICA) University of Cádiz Cádiz Spain; 2 Department of Nursing and Physiotherapy University of Cádiz Cádiz Spain; 3 The University Research Institute for Sustainable Social Development (INDESS) Jerez de la Frontera Spain; 4 See Acknowledgements Cádiz Spain

**Keywords:** complex health needs, health literacy, heart failure, mHealth, multimorbidity

## Abstract

**Background:**

Patients with multimorbidity and complex health needs are defined as a priority by the World Health Organization (WHO) and the European Union. There is a need to develop appropriate strategies with effective measures to meet the challenge of chronicity, reorienting national health systems. The increasing expansion of mobile health (mHealth) interventions in patient communication, the reduction of health inequalities, improved access to health care resources, adherence to treatment, and self-care of chronic diseases all point to an optimistic outlook. However, only few mobile apps demonstrate their effectiveness in these patients, which is diminished when they are not based on evidence, or when they are not designed by and for users with different levels of health literacy (HL).

**Objective:**

This study aims to evaluate the efficacy of an mHealth intervention relative to routine clinical practice in improving HL and self-management in patients with multimorbidity with heart failure (HF) and complex health needs.

**Methods:**

This is a randomized, multicenter, blinded clinical trial evaluating 2 groups, namely, a control group (standard clinical practice) and an intervention group (standard clinical practice and an ad hoc designed mHealth intervention previously developed), for 12 months.

**Results:**

The contents of the mHealth intervention will address user-perceived needs based on the development of user stories regarding diet, physical exercise, cardiac rehabilitation, therapeutic adherence, warning signs and symptoms, and emotional management. These contents have been validated by expert consensus. The creation and development of the contents of the mHealth intervention (app) took 18 months and was completed during 2021. The mobile app is expected to be developed by the end of 2022, after which it will be applied to the experimental group as an adjunct to standard clinical care during 12 months.

**Conclusions:**

The trial will demonstrate whether the mobile app improves HL and self-management in patients with HF and complex health needs, improves therapeutic adherence, and reduces hospital admissions. This study can serve as a starting point for developing other mHealth tools in other pathologies and for their generalization to other contexts.

**Trial Registration:**

ClinicalTrials.gov NCT04725526; https://tinyurl.com/bd8va27w

**International Registered Report Identifier (IRRID):**

DERR1-10.2196/35945

## Introduction

### Care of Patients With Multimorbidity and Heart Failure

Although increased life expectancy, socioeconomic improvements, and biomedical innovations have led to a reduction in mortality, they have also led to a considerable increase in chronic conditions. A prevalent paradigm of these socio-health phenomena is the multimorbidity of patients, which is one of the leading health problems of the 21st century. Multimorbidity can alter health outcomes and lead to disability that is more significant or can lead to worse quality of life or frailty [1].

Heart failure (HF) has a high and increasing prevalence and incidence, and is one of the leading causes of morbidity and mortality in the Western world [2]. Further, if we add to this the significant impact of chronic diseases, mainly cardiovascular diseases, cancer, diabetes, and chronic lung diseases [3], the situation becomes even more complicated by the fact that patients are responsible for most of the avoidable hospital admissions. In this line, both the World Health Organization (WHO) and the European Union define a patient with chronic disease as a priority and point out the need to develop appropriate strategies with effective measures to meet the challenge of chronicity, reorienting national health systems [4,5].

According to the latest data published by the WHO [4], heart disease continues to be the leading cause of mortality worldwide. Globally, it was estimated that in 2019, 197.2 million people were living with ischemic heart disease (IHD), and it was more prevalent in males than in females (113.7 and 83.6 million people, respectively). North Africa and the Middle East, Central Asia, and Eastern Europe had the highest prevalence rates of IHD in the world. The number of deaths due to heart disease has increased since 2000 by more than 2 million people, from 6-7 million to almost 9 million people, representing 16% of all deaths. In 2019, IHD mortality rates were 118.0 per 100,000. IHD mortality rates were highest in parts of North Africa and the Middle East, Eastern Europe, and Central Asia [6,7].

In Spain, HF remains an enormous health challenge (estimates suggest that there are more than 1,300,000 people with HF). Besides, more than 17,000 people die from this disease every year, making it the fourth leading cause of death [8] and the third leading cause of cardiovascular death after IHD and cerebrovascular disease. It is also the leading cause of hospitalization in people over 65 years of age; furthermore, 50% die within 5 years of diagnosis. The prevalence of HF increases progressively with age, reaching prevalence rates of 1%, 10%, and 17.4% in the population over 40, 70, and 85 years of age, respectively. There are more than 80,000 admissions per year in Spain for HF, and half of the hospitalized patients are readmitted within 1 year due to decompensation. Likewise, the use of pharmacological and nonpharmacological resources (resynchronizers or defibrillators) in people with HF is growing exponentially [9].

Although there is a growing interest in patients with comorbidity and multimorbidity, from an evidence perspective, this population has been excluded from most clinical trials and intervention studies [10,11]. Interdisciplinary teams based on the collaboration of different care settings and sustainable interventions adjusted to the public system are recommended. Similarly, a model centered on the patient’s needs is reinforced, based on primary care and shared action with hospital care, with proactive and planned interventions aimed at promotion and prevention [12].

### Health Literacy as a Health Asset in Patients With Multimorbidity

In the field of public health and health promotion, health literacy (HL) is presented as a means that allows individuals to exercise significant control over their health and over the personal, social, and environmental determinants that determine it, being considered as an individual asset to be built.

Following the theoretical framework proposed by the European Health Literacy Project (European Health Literacy Survey [HLS-EU]) [13], HL is determined by the combination of the 3 dimensions of health (being sick/health care; being at risk/disease prevention, and being healthy/health promotion) and the 4 ways of managing information (finding it, understanding it, evaluating it, and applying it to one’s own life to make informed decisions). *Access* refers to the ability to seek, find, and obtain health information; *understanding* refers to the individual’s ability to understand the info accessed; *evaluation* describes the ability to interpret, filter, judge, and assess the health information accessed and understood; and *application* refers to the ability to communicate and use the information to decide to maintain and improve health.

Populations that are more likely to experience difficulties in self-managing their diseases are those with low levels of HL [14]. Having poor HL is an independent risk factor for poorer health [15] because of medication errors and a poorer understanding of disease and treatments [16]. Similarly, there is evidence of a relationship between low HL and higher rates of hospital admissions, poorer therapeutic adherence on care plans, and poor use of preventive services [17,18]. In the particular case of people with chronic diseases, HL plays a crucial role in the self-management of their disease [19].

### mHealth: A Digital Health Literacy Proposal

In the last few years, mobile health (mHealth) has emerged prominently as a result of the tremendous sociological and cultural impact of smartphones and tablets.

The Global Observatory for eHealth defines it as “medical and public health practice carried out with the support of mobile devices such as phones, personal digital assistants, Tablets and other wireless communication devices to carry out public health activities and assist in clinical practice” [20].

Despite its expansion in recent years, few studies demonstrate the effectiveness and utility of mHealth in chronic disease self-management beyond diabetes [21,22]. A recent systematic review of clinical trials involving mHealth interventions to improve self-management of patients with chronic diseases, including patients with cancer, HF, fibromyalgia, asthma, and spine bifida, achieved statistically significant effects on health outcomes after the incorporation of mobile apps in disease management [23]. Existing evidence promises an optimistic horizon regarding their effectiveness in chronic diseases [23,24] and, in particular, in patients with cardiac diseases [25,26]. Despite this, we still find contradictory results. For example, the study by Hägglund et al [27] in patients with HF revealed improvements in self-care and quality of life and a reduction in the number of hospitalization days. By contrast, the study by Vuorinen et al [28] found no difference in the number of hospitalization days.

At present, there is a severe deficiency of mHealth tools, which are developed in collaboration with target patients and multidisciplinary teams, are incorporated into daily care practice, and have proven their efficacy in clinical trials [29,30], to improve the self-management of patients with HF having complex health problems. Systematic reviews recently published on this topic [23,25,26] reflect the scarcity of studies aimed at patients with multimorbidity.

Therefore, we aim to develop an app based on the needs of users and deficiencies identified by professionals, which motivates behavior change through gamification strategies, as per scientific evidence and adapted to the user’s level of HL, and to test its effectiveness in terms of self-management and improvement of personal autonomy to perform basic activities of daily living, reduce hospital admissions, promote therapeutic adherence, and increase HL.

## Methods

### Study Design

A randomized, controlled, multicenter clinical trial evaluated the efficacy of an mHealth intervention with 2 groups: a control group (standard clinical practice) and an experimental group (standard clinical practice together with an ad hoc designed mHealth intervention).

### Patient Selection

#### Setting

A total of 4 basic health areas located in the south of Spain, including 2 hospitals, will participate. The health care centers belonging to the clinical management units with the best mortality rates during hospital admission and at 30 days after discharge (12.7% and 14.3 %, respectively) and those with the highest rates (28% and 30.4%, respectively) were considered.

#### Participants

Patients with multimorbidity with HF and complex health need to be attended by the nurse case manager of the primary care or hospital care centers, nurse or family doctor, or specialized care nurse, or area specialist physician of the study area.

#### Diagnostic Criteria

Patients with multimorbidity and HF and complex health needs who meet the following diagnostic criteria [12] will be considered:

- Be classified in clinical category A of chronic pathologies for HF that, in a situation of clinical stability, has been in grade II of the New York Heart Association (NYHA) [31], being able to be simultaneously classified, or not, in other clinical categories for having another chronic disease(s) included in these categories.
- Patients with at least one of the following complexity criteria [12]: extreme polypharmacy (≥10 chronically prescribed active ingredients); sociofamilial risk (Gijon scale score [32] >10 points); pressure ulcers of stage II or higher; malnutrition (body mass index <18.5 kg/m^2^); nasogastric feeding (≥3 months); 2 or more hospital admissions in the previous 12 months.

#### Inclusion Criteria

The following inclusion criteria will be applied:

- patients of both sexes over 18 years of age;
- patients attended by health care professionals of the basic health areas participating in the study;
- patients who give their agreement to participate in the study by signing an informed consent form;
- patients who have a mobile device (smartphone or tablet) compatible with the Android or iOS operating system;
- considered as a patient with multimorbidity based on the criteria described in the previous section.

#### Exclusion Criteria

The following exclusion criteria will be applied:

- patients with sensory deficits or upper limb mobility problems that prevent them from using the app correctly, despite using the accessibility features on mobile devices;
- patients with persistent cognitive impairment (Pfeiffer [33] with ≥5 errors or Lobo Mini-Cognitive Test score <23 [34]) or severe mental disorder;
- patients with severe limitations for basic activities of daily living (Barthel Index <20 points [35]).

#### Eligibility Criteria

Meet all inclusion criteria and none of the exclusion criteria.

#### Completion Criteria and Withdrawal

A patient will be considered to have completed the study when he/she completes the postintervention evaluation. The criteria for withdrawal of a participant will be

- failure to receive the complete intervention (unsolvable technical failures in the device) or by the protocol (failure to use the app);
- loss of compliance with the eligibility criteria during the study or withdrawal of consent to participate;
- inability to contact for follow-up.

#### Sample Size

The HL will be selected as the main outcome to evaluate the effectiveness. This outcome will be measured using the Spanish version of the 47-Item European Health Literacy Questionnaire (HLS-EU-Q47), which achieved a mean score of 32.88, over a possible range of 0 to 50 points, with an SD of 6.10 in the study setting [36]. An increase of at least 10% in the score will be considered clinically relevant due to the mHealth intervention. Accepting an α risk of .05 and a β risk of .90 in a bilateral contrast, 118 participants were needed in every group. A loss to follow-up rate of 30% was considered. However, the calculation is based on a minimum reference of sample observations, so an effort will be made to recruit as many participants as possible to increase the precision of the estimates.

### Intervention

#### Design of the mHealth App

For the design of the mHealth app, the expert consensus and the modified Delphi will be used. The consensus conference will consist of 12 experts, with 2 representatives having each of the following profiles: (1) health care professional with experience (>5 years) in the care of patients with multimorbidity or HF; (2) university teaching and research staff with experience in research projects in the thematic areas addressed (HL; intervention programs, or patients with multimorbidity); (3) other professionals with experience in research, assistance, or care of patients with multimorbidity (social workers, psychologists, communication professionals); (4) computer engineers with experience in the design of information and communication technology tools; (5) representatives of associations of patients with chronic diseases; and (6) patients with multimorbidity and HF. The consensus sessions will incorporate the main agreements adopted regarding the contents of the intervention program, format, logistical coordination for the development of the program, and proposals for sustainability. Once consensus has been reached, the contents will be established together with the graphic script of the future tool. Delphi rounds will validate the content of the wireframes and the storyboard in terms of relevance (significance of the content for the objective of the intervention program) and appropriateness (content fit). The optimal size for a Delphi group is estimated to be between 6 (minimum) and 30 (maximum) [37]. Considering the response/abandonment rate, we will try to tend to the maximum number of experts who will be different from those that participated in the consensus conference but will meet the same criteria. A 4-point Likert scale (1=no relevance/adequacy and 4=high relevance/adequacy) will be included with each content screen. The experts will be required to evaluate the content’s relevance and appropriateness in each screen. The Content Validity Index (CVI) [38] of each screen will be calculated.

Similarly, the Adequacy Index (AI) will be analyzed. The relevance/appropriateness of the screens will be considered good if the CVI and AI are greater than or equal to 0.78 [38]. Those screens that do not reach the preset value in the aforesaid indices will be reviewed and reformulated. Screens’ content will also be modified based on the feedback collected and subjected to the same process described above until a final version is agreed upon. This prototype will be sent to a developer to create the mHealth tool under an agile approach. This process (designing, creating, and developing the mobile app) will take 18 months.

#### Description of the Intervention

The mHealth app aimed at empowering HL and self-management of the patients with multimorbidity with HF and complex health needs. It will be developed ad hoc based on the information obtained from the consensus sessions of experts (patients and professionals) and in accordance with the gamification methodology [39]. Although the final choice of the content will depend on the resulting decisions of the experts, it is intended to offer information and resources to the patient according to the level of HL identified and considering the main actions recommended to be incorporated to improve the overall health outcomes of these patients [12]. Thus, the participants in the experimental group will receive the mHealth app and will agree with the health care professional on 3 objectives related to the self-management of their disease, which they will have to achieve within a proposed period, being assisted by the mobile app, which will provide feedback and reinforcement in the achievement of these objectives. The stages and timing are described in Table 1. The intervention carried out in the control group participants will be based on usual clinical practice. Like the experimental group, they will agree with the health professional on 3 objectives they will have to achieve within the proposed time frame. This process (evaluation of the efficacy of the mHealth intervention with 2 groups) will last 12 months.

**Table 1:** Stages of intervention.

Stage	Description	Timing (months)												
0	Detection of potential study participants. Presentation of the project and collection of informed consent.	✓^a^												
1	Baseline data collection for both groups.	✓												
2	For both groups, the patient and the practitioner will agree on the first health goal. On an individual basis, the nurse will present the main aspects related to the use and management of the app to the members of the expert group. The patient’s expectation of self-efficacy in using the app will be assessed to prevent adherence problems concerning the use of mobile health and difficulties in using the app (digital literacy) will be resolved.	✓	✓	✓										
3	The patient will come for a consultation to check compliance with the chosen objective. If necessary, a simulation test will be performed to confirm that the challenge has been met. At the end of this stage, the patient and the multidisciplinary team will agree on the second objective.			✓	✓	✓	✓							
4	Check compliance with the second objective. Perform simulation test, if required. At the end of this stage, the patient and the multidisciplinary team will agree on the third objective.						✓	✓	✓	✓				
5	Check compliance with the third objective. Perform simulation test, if required.									✓				
6	Postintervention evaluation: all health objectives were achieved by the patient and postintervention evaluation is performed										✓	✓	✓	
7	Follow-up	At 12 months after the intervention												

^a^✓: achievement record.

### Process

#### Assessment Eligibility

The health care professionals participating in the study will verify that the patient meets the eligibility criteria by consulting the digital history and interviewing the patient.

#### Randomization

Block randomization has been considered. The total number of participants will be divided by the number of study centers in equal parts. The number assigned will be for each center and will be considered a block in terms of randomization. An external collaborator (blinded) will use a computer-generated randomization list to assign patients to the groups such that, in each block (center), 50% of the patients will go to the control group (usual clinical practice) and 50% to the intervention group (usual clinical practice and mHealth app). This information will be available only to the principal investigator.

#### Masking

Because of the characteristics of the study, it is not possible to blind it to the participants or to the researchers who will carry out the intervention. However, it will be masked to the researchers who will perform the effectiveness evaluation and data analysis. For this purpose, the coding of the randomization variable will be hidden from them and will be guarded by a person outside the project selected by the principal investigator. In this way, it will not be possible to know which groups received the intervention until the analyses are completed.

#### Sample Recruitment

Contact with the participants will be established through consultations with the nurse case manager of the basic primary care team of each participating center or the consultations with the hospitals belonging to the scope of the study and patient associations. Once they have ensured that the patient meets the eligibility criteria, the patient will be invited to join the study.

### Evaluation of Effectiveness

#### Sample Characterization, Primary and Secondary Assessment Variables, and Data Source.

See summary in Tables 2 and 3.

**Table 2:** Measurement, recording, and data source of variables of characterization.

Type of variable		Register rank/format	Measurement	Source of data
**Characterization: sociodemographic**				
	Sex	Male Female	Registration	Medical history
	Age	18-99	Registration	Medical history
	Marital status	Single Married Widower Divorced/separated	Registration	Registration
	Nationality	Spanish European Union Rest of Europe North America Latin America Africa Asia Other	Registration	Registration
	Education level	No education Primary school Secondary school University degree Master’s degree Doctorate	Registration	Registration
	Occupation	Employed Self-employed Unemployed Retired Permanently disabled Homemaker Student Other	Registration	Registration
**Characterization: clinical**				
	Main diagnosis	ICD-9-CM^a^ codes: 402.01 402.11 402.91 404.01 404.11 409.91 428.x	Registration	Medical history
	Years since diagnosis	0-100	Registration	Medical history
	Other secondary chronic pathologies	ICD-9-CM codes	Registration	Medical history
	HF^b^ signs/symptoms (dyspnea, orthopnea, fatigue, edema, oliguria, paroxysmal nocturnal dyspnea, high venous pressure, crackles R3/R4, murmurs and hepatomegaly)	Yes No	Registration or exploration or anamnesis	Medical history
	Cardiovascular risk factors (smoking, diabetes mellitus II, obesity [weight/BMI], hypertension, previous heart disease, dyslipidemia)	Yes No	Registration or exploration or anamnesis	Medical history
	HF functional classification (establishes the functional severity of HF based on stress tolerance)	I: No limitation. Regular physical activity does not cause dyspnea, fatigue, or palpitations. II: Slight limitation. Usual physical activity causes dyspnea, fatigue, or palpitations. III: Marked limitation (minor activities cause symptoms). IV: Inability to perform any activity. Symptoms even at rest.	NYHA^c^ Functional Classification [31]	Registration medical history

^a^ICD-9-CM: International Classification of Diseases, 9th revision, Clinical Modification.

^b^HF: heart failure.

^c^NYHA: New York Heart Association.

**Table 3:** Measurement, recording, and data source of variables of main and secondary responses.

Type of variable		Register rank/format	Measurement	Source of data
**Of main response**				
	Health literacy	0/50	HLS-EU-Q47^a^ [36]	Registration
		1-5/indicator	NOC^b^ code: 2015 [29]	Nursing record
	Self-management	1-5/indicator	NOC codes: 3102; 1803; 1830 [9,40]	Nursing record
		12-60	European Heart Failure Self-Care Behavior Scale [41]	Nursing record
	Number of readmissions 1m;12m	0-99	Registration	Registration medical history
**Secondary response**				
	Personal autonomy basic activities of daily living	0-100	Barthel Index [35]	Registration
	Personal autonomy instrumental activities of daily living	0-8	Lawton-Brody Index [42]	Registration
	Therapeutic adherence	0-4	Morisky-Green Adaptation Questionnaire [43] and Recipe XXI	Registration
	Vital forecast	0-30	PROFUND Index [44]	Registration
	Satisfaction with service	0-10	Satisfaction Scale	Nursing record
	Mobile health device satisfaction	1-5	Satisfaction Scale	Built-in mobile health device registration

^a^HLS-EU-Q47: 47-item European Health Literacy Questionnaire.

^b^NOC: Nursing Outcomes Classification.

#### Security Variables

No adverse effects of patients’ exposure in the experimental group were considered, so no safety variables were included in the study.

#### Data Collection and Custody

Participating nurses and physicians will be responsible for data recording during all stages of the intervention (before training) and the collection and custody of informed consent (Multimedia Appendix 1). A password-coded data file will be created for researchers for the collection of a case. The necessary technical and logistical means will be established to ensure that the processing, communication, and transfer of personal data of all participants comply with the provisions of Organic Law 15/1999 of December 13 to protect personal data [45].

### Data Analysis: Data Encoding

A data matrix will be created, and data will be processed statistically using SPSS, version 22 (IBM). Statistical significance will be set at 95% (α=.05).

A uni-bivariate descriptive analysis will be performed to determine the sample distribution for each of the variables studied, both for the total sample and for each group. The characterization variables will be summarized using descriptive statistics, expressing qualitative variables in terms of frequency and percentages and quantitative variables in terms of mean and SD.

Before the analysis, the normality of the variables will be evaluated using the Kolmogorov-Smirnov and Shapiro-Wilk tests. Baseline differences in the variables related to the sample profile between the intervention and control groups will be compared using the Student *t* test when these are normally distributed and the nonparametric Mann-Whitney *U* test in case they are not. Differences between dichotomous qualitative variables will be established using the chi-square test or Fisher statistic when appropriate.

A contrast of means will be applied to test the efficacy of the intervention in terms of the normality distribution of the variables. The results will be analyzed using the nonparametric Wilcoxon signed-rank test, which will allow us to check if there are differences between 2 populations from 2 dependent or related samples, or the Student *t* test for normally distributed samples. For independent samples, the nonparametric Mann-Whitney *U* test will be used.

The strength of the relationship between continuous data will be determined from the Pearson or Spearman correlation. The intraclass correlation coefficient will be used if it is necessary to measure concordance between measurements at different times of the study. To identify sociodemographic and clinical characterization variables (independent variables) related to the intervention and the different response variables (dependent variables), multivariate, linear, logistic, or Cox proportional analyses will be performed, as appropriate for each response variable.

### Ethics and Dissemination

This study has been approved by the Cadiz Research Ethics Committee. The aim of the study and the anonymity of participants, as well as the voluntary nature of participation, will be explained before the participants start and their informed consent will be requested. The participants will also be informed that the data obtained would be used for research purposes only. Findings from this study will be disseminated through peer-reviewed scientific journals and at key national and international scientific events. The study was registered in ClinicalTrials.gov (trial registration number: NCT04725526) on January 26, 2021.

### Public/Patient Involvement Statement

Neither the patients nor the general public were involved in the design, or conduct, or reporting, or dissemination plans of the study.

## Results

The content validation to develop the mobile app was completed in 2021. To optimize material and human resources, the research team has been divided into 6 working subgroups that correspond to the main areas of action for patients with multimorbidity with HF: (1) physical exercise and cardiac rehabilitation, (2) nutrition, (3) medication and therapeutic adherence, (4) warning signs and symptoms, (5) self-care/self-management (which includes the elimination of toxic habits), and (6) emotional management.

Throughout the first year of the project, comprehensive/systematic bibliographic reviews of the scientific evidence on mHealth interventions (by the thematic areas described above) aimed at improving the evolution/care of patients with multimorbidity with HF have been carried out.

Individualized telephone interviews with patients were conducted to obtain information on their opinions, needs, and experiences about the disease management. This information has been incorporated into the objectives/contents of the mHealth app. Based on these data, an expert consensus (professionals and patients) has been conducted to establish the contents and structure of the mobile app to proceed with the content validation and its beta version.

The results of the primary and secondary variables recorded in the experimental group (health literacy, number of readmissions, self-management, therapeutic adherence, general satisfaction, and performance in activities of daily living) will improve and there will be significant differences with the control group. The findings will be disseminated to stakeholders using various methods, including peer-reviewed journals, academic conferences, and other verbal and digital communication channels. In relation to the patentability of project results, the resulting mHealth app will be registered as an intellectual property.

## Discussion

### Study Summary

To our knowledge, this is the first study to develop a protocol for a randomized controlled trial of an mHealth intervention to improve HL and self-management in patients with multimorbidity with HF and complex health needs that is based on patients’ perceived needs. The content of the intervention has already been validated.

We will investigate the efficacy, effectiveness, and usability of the proposed intervention in patients with multimorbidity and HF. The experimental group will use the app, hoping that the results of the primary and secondary variables recorded will improve and that significant differences will become evident after use and after comparison with the control group.

It is expected that the intervention developed will be effective and, therefore, improve the level of HL, self-management and therapeutic adherence, reduce the number of admissions per year due to decompensation, and that the incidence rates of HF mortality (adjusted for age, sex, and risk) or 30-day postdischarge mortality of the experimental group under study will be lower than that of the control group.

### Limitations

This study has some limitations that should be acknowledged. The lack of adequate mobile devices or the sensory or cognitive deficits of the target population is taken into account. Likewise, their digital illiteracy could be another limitation. Therefore, and being aware of the population group, digital training is contemplated. As for cognitive impairment, the usual caregiver is included in the intervention.

When evaluating the effectiveness of the intervention, we can find the influence of the variable “time of use of the app” (a record of access to the app will be obtained and monitored).

Finally, we must be aware of the possible loss of participants due to their clinical characteristics. Therefore, when determining the sample size, a loss rate of 30% has been taken into account. The aim is to obtain as many participants as possible to increase the precision of the estimates.

### Strengths

With the aim of overcoming the aforementioned limitations, a qualitative analysis of the risks has been carried out and has been prioritized according to the factor of exposure and urgency.

Randomized controlled trials are considered the “gold standard for assessing efficacy in clinical research and constitute evidence for treatment” [46]. By adopting such a trial and ensuring internal and external validity, we maximized the robustness of our study.

In addition, the multidisciplinary nature of this project is evident in the composition of the research team (psychologist, nurses, physicians, computer engineers), in the incorporation of professionals and patients for its development, and in the association of the 2 public organizations involved in it.

Finally, the cross-cutting nature of this project is reflected in the consideration of the different dimensions of health and the different levels of health care (primary and hospital care). By contrast, the main variable of this project (HL) has a cross-cutting nature as it has been recognized as a basic social determinant for improving health outcomes.

### Conclusions

The results of this intervention support the coordinated work between hospital care and primary care, which will have an impact on the improvement of health care and management, thus favoring the continuity of care and the reduction of hospital readmissions in this population.

The incorporation of the mobile app developed will optimize the work performed by professionals while increasing patients’ HL and reducing the number of consultations requested and, ultimately, health care costs.

This study can serve as a starting point for developing other mHealth interventions in other pathologies and for their generalization to other contexts.

## Appendix

Multimedia Appendix 1 Informed consent.

Multimedia Appendix 2 Peer-Review report from El Instituto de Investigación e Innovación en Ciencias Biomédicas de la Provincia de Cádiz (INiBICA) - Fundación Biomédica Cádiz - Proyectos Investigacion Innovacion (Cádiz, Spain).

## Abbreviations

AI: Adequacy Index
CVI: Content Validity Index
HF: heart failure
HL: health literacy
HLS-EU: European Health Literacy Survey
HLS-EU-Q47: 47-item European Health Literacy Questionnaire
ICD-9-CM: International Classification of Diseases, Ninth Revision, clinical modification
IHD: ischemic heart disease
mHealth: mobile health
NOC: Nursing Outcomes Classification
NYHA: New York Heart Association
WHO: World Health Organization
