# Correction: Development and Effectiveness of a Mobile Health Intervention in Improving Health Literacy and Self-management of Patients With Multimorbidity and Heart Failure: Protocol for a Randomized Controlled Trial

**DOI:** 10.2196/44570

**Published:** 2022-12-07

**Authors:** Pilar Bas-Sarmiento, Martina Fernández-Gutiérrez, Miriam Poza-Méndez, Antonio Jesús Marín-Paz, Olga Paloma-Castro, José Manuel Romero-Sánchez, ASyAG_PPIC Team

**Affiliations:** 1 Institute of Research and Innovation in Biomedical Sciences of the Province of Cádiz (INiBICA) University of Cádiz Cádiz Spain; 2 Department of Nursing and Physiotherapy University of Cádiz Cádiz Spain; 3 The University Research Institute for Sustainable Social Development (INDESS) Jerez de la Frontera Spain; 4 See Acknowledgements

In “Development and Effectiveness of a Mobile Health Intervention in Improving Health Literacy and Self-management of Patients With Multimorbidity and Heart Failure: Protocol for a Randomized Controlled Trial” (JMIR Res Protoc 2022;11(4): e35945), the authors noted one error.

In the originally published article, one of the members of the ASYAG_PPIC Team’s name incorrectly appeared under the Acknowledgments section as:

José Crespo-Piñero

It has now been replaced by:

José Castro-Piñero

The final Acknowledgments section will appear as follows:

This study has received financial support from The Institute of Research and Innovation in Biomedical Sciences of the Province of Cadiz. The initial protocol has undergone peer review by the funding body. The funding body supervises the conduct of the overall project but is not involved in any operations. The authors are responsible for the execution, content, and results of the materials. The ASyAG_PPIC Team (Institute of Research and Innovation in Biomedical Sciences of the Province of Cádiz [INiBICA], University of Cádiz, Cádiz, Spain) consist of: José María Cano-Guerrero; Inés Carmona-Barrientos; María Ángeles Carrasco-Bernal; Mónica Casado-Daza; José Castro-Piñero; Cristina Castro-Yuste; Magdalena Cuenca-García; Ignacio DelARco-Herrera; Pedro Díaz-deSouza; Mercedes Díaz-Rodríguez; María Falcón-Romero; Jorge del Rosario Fernández-Santos; Laura Gallardo-Amaro; María Paz Gómez-Jiménez; Gloria González-Medina; Eulalia Hernández-Encuentra; Luis Javier Moreno-Corral; Petronila Oliva-Ruiz; Francisco Javier Ordoñez-Muñoz; Ceferino Prieto-García; Inmaculada Ramón-Macías; Manuel Rosety-Rodríguez; Víctor Segura-Jiménez; María Jesús Viñolo-Gil; Juan Carlos Paramio-Cuevas; Mercedes Ruiz-Carreira; Eduardo Sánchez-Sánchez; Alezandra Torres-Castaño; Javier María Yagüe-Sánchez.

The correction will appear in the online version of the paper on the JMIR Publications website on December 7, 2022, together with the publication of this correction notice. Because this was made after submission to PubMed, PubMed Central, and other full-text repositories, the corrected article has also been resubmitted to those repositories.

