# Supplementary material for: Development and Effectiveness of a Mobile Health Intervention in Improving Health Literacy and Self-management of Patients With Multimorbidity and Heart Failure: Protocol for a Randomized Controlled Trial
Source: JMIR Res Protoc. 2022 Apr 29;11(4):e35945. doi: 10.2196/35945 (PMC9107042; doi:10.2196/35945)
Supplement: Multimedia Appendix 1 [file resprot_v11i4e35945_app1.docx]

**Anexo 1. Hoja de Información al Paciente**

| **TÍTULO DEL ESTUDIO** | Desarrollo y efectividad de una intervención de mHealth en la mejora de la Alfabetización en Salud y autogestión del paciente pluripatológico con insuficiencia cardíaca: un ensayo controlado aleatorizado. |
| --- | --- |
| **CENTRO** | Universidad de Cádiz/Área de Gestión Sanitaria Campo de Gibraltar |

Nos dirigimos a usted para informarle sobre un estudio de investigación en el que se le invita a participar. El estudio ha sido aprobado por el Comité Ético de Investigación de Cádiz con fecha________________.

Nuestra intención es que usted reciba la información correcta y suficiente para que pueda decidir si quiere o no participar en este estudio. Para ello lea esta hoja informativa con atención y le aclararemos las dudas que le puedan surgir después de la explicación. Además, puede consultar con las personas que considere oportunas.

**PROPÓSITO DEL ESTUDIO**

El estudio consiste en evaluar la eficacia de una intervención de mHealth, como complemento a la práctica clínica habitual, respecto a la práctica clínica exclusivamente. Queremos saber si la aplicación móvil que vamos a facilitarles hace que los pacientes estén más informados, comprendan mejor la información necesaria para manejarse eficazmente y tomen decisiones de salud informadas y mejoren, en definitiva, sus resultados de salud.

**PROCEDIMIENTO**

Utilizando un sistema de asignación aleatoria, usted puede ser asignado/a por azar al grupo de pacientes a los que se les facilite la aplicación móvil o al grupo de pacientes que siga el tratamiento habitual. Por tanto, la posibilidad de ser asignado/a a uno u otro grupo es del 50%. Nos gustaría señalar que su participación es igual de valiosa para el estudio independientemente del grupo al que sea asignado.

En el caso de que sea seleccionado para el grupo que utilice la aplicación móvil, en la consulta de enfermería le explicaran con todo detalle cómo funciona y qué recomendaciones debe seguir. Considere que ha sido desarrollada teniendo en cuenta la información de pacientes que han pasado por esta misma situación, así como profesionales expertos en enfermedades crónicas y patología cardiaca. Deberá usar dicha aplicación durante un mes y visitar a su enfermera cada 15 días durante dicho periodo.

Independientemente del grupo al que sea asignado/a, el personal sanitario de su centro realizará una valoración integral de su estado y grado de conocimiento, comprensión y manejo de la enfermedad. Además, por el hecho de participar, usted acepta que nos pongamos en contacto con usted pasado un año para hacerle una breve entrevista telefónica sobre su experiencia y estado de salud.

**BENEFICIOS Y POSIBLES RIESGOS**

Existen estudios previos que determinan una mejora en el manejo de la enfermedad que usted padece y en la reducción del número de días de hospitalización tras el uso de una aplicación móvil. Sin embargo, hay otros estudios que no han podido confirmar dichos hallazgos. Es por ello que **se desconoce si obtendría algún beneficio para su Salud** por participar en este estudio.

Las investigaciones realizadas hasta el momento utilizando dispositivos de mhealth en pacientes en otro tipo de contextos no especifican ninguna posible complicación por su uso. No obstante, deberá comunicar a los profesionales que le atienden cualquier efecto negativo que usted considere que puede estar experimentado durante su uso.

**PARTICIPACIÓN VOLUNTARIA Y RETIRADA DEL ESTUDIO**

Debe saber que su participación en este estudio es voluntaria y que puede decidir NO participar sin consecuencias en la atención que va a recibir. Si decide participar, puede cambiar de opinión y retirar su consentimiento en cualquier momento, sin que por ello se altere la relación con los profesionales sanitarios que le atienden ni se produzca perjuicio alguno en su atención sanitaria.

**CONFIDENCIALIDAD**

El estudio cumplirá lo establecido en la Ley Orgánica 15/1999, de 13 de diciembre de protección de datos de carácter personal y al Real Decreto que la desarrolla (RD 1720/2007). Los datos recogidos para el estudio estarán identificados mediante un código, de manera que no incluya información que pueda identificarle. El tratamiento, la comunicación y la cesión de los datos de carácter personal de todos los participantes se ajustarán a lo dispuesto en esta ley.

**A QUIÉN CONTACTAR**

Si requiere información adicional o desea ejercer sus derechos de acceso, rectificación, consulta u oposición de los datos, puede ponerse en contacto con la Investigadora Principal del proyecto Dra. Pilar Bas Sarmiento o Dra. Martina Fernández Gutiérrez en el teléfono 856028100 o en el correo electrónico: [pilar.bas@uca.es](mailto:pilar.bas@uca.es); [martina.fernandez@uca.es](mailto:martina.fernandez@uca.es)

**Hoja de Consentimiento de Participante/CONSENTIMIENTO INFORMADO**

| **TÍTULO DEL ESTUDIO** | Desarrollo y efectividad de una intervención de mHealth en la mejora de la Alfabetización en Salud y autogestión del paciente pluripatológico con insuficiencia cardíaca: un ensayo controlado aleatorizado. |
| --- | --- |
| **CENTRO** | Universidad de Cádiz/Área de Gestión Sanitaria Campo de Gibraltar |

Yo, *__________________________________________________________________________*

⎕ He leído la hoja de información que se me ha entregado sobre el estudio.
⎕ He podido hacer preguntas sobre el estudio.
⎕ He recibido suficiente información sobre el estudio.
⎕ He hablado con __________________________________________.
⎕ Comprendo que mi participación es voluntaria.
⎕ Comprendo que puedo retirarme del estudio:
 **-** Cuando quiera.
 - Sin tener que dar explicaciones.
 - Sin que esto repercuta en mis cuidados de salud / médicos.

⎕ Recibiré una copia firmada y fechada de este documento de consentimiento informado
⎕ Presto libremente mi conformidad para participar en el estudio.

Deseo que me comuniquen la información derivada de la investigación que pueda ser relevante para mí salud:

⎕ SÍ

⎕ NO

Firma del participante Firma del investigador Fecha: ____/____/____ Fecha: ____/____/____ (Nombre, firma y fecha de puño y letra por el paciente)

Teléfono de contacto 1 __________________________________________________________

Teléfono de contacto 2 __________________________________________________________

Correo electrónico contacto ______________________________________________________

**Hoja de Consentimiento de Participante/CONSENTIMIENTO ORAL ANTE TESTIGOS**

| **TÍTULO DEL ESTUDIO** | Desarrollo y efectividad de una intervención de mHealth en la mejora de la Alfabetización en Salud y autogestión del paciente pluripatológico con insuficiencia cardíaca: un ensayo controlado aleatorizado. |
| --- | --- |
| **CENTRO** | Universidad de Cádiz/Área de Gestión Sanitaria Campo de Gibraltar |

Yo, *__________________________________________________________________________*

⎕ He leído la hoja de información que se me ha entregado sobre el estudio.
⎕ He podido hacer preguntas sobre el estudio.
⎕ He recibido suficiente información sobre el estudio.
⎕ He sido informado por __________________________________________.

⎕ Comprende que su participación es voluntaria.
⎕ Comprende que puede retirarme del estudio:
 **-** Cuando quiera.
 - Sin tener que dar explicaciones.
 - Sin que esto repercuta en sus cuidados de salud / médicos.

Y ha expresado libremente su conformidad para participar en el estudio.

Firma del testigo
Fecha: ____/____/____

Teléfono de contacto 1 __________________________________________________________

Teléfono de contacto 2 __________________________________________________________

Correo electrónico contacto ______________________________________________________
