# Supplementary material for: Development and Effectiveness of a Mobile Health Intervention in Improving Health Literacy and Self-management of Patients With Multimorbidity and Heart Failure: Protocol for a Randomized Controlled Trial
Source: JMIR Res Protoc. 2022 Apr 29;11(4):e35945. doi: 10.2196/35945 (PMC9107042; doi:10.2196/35945)
Supplement: Multimedia Appendix 2 [file resprot_v11i4e35945_app2.pdf]

MINISTERIO
DE CIENCIA, INNOVACIÓN
Y UNIVERSIDADES

AGENCIA ESTATAL DE INVESTIGACIÓN

Informe evaluación final Fundación Biomédica Cádiz PROYECTOS INVESTIGACION INNOVACION (INIBICA) 2019

**Referencia:**	INBICA2019-18	**Fecha:**	26/11/2019 08:49:59
**Área:**	*PSI-Psicología		
**Subárea:**	PSI		
**Solicitantes**	Bas Sarmiento, Pilar		
**Título:**	Desarrollo y efectividad de una intervención de mHealth en la mejora de la Alfabetización en Salud y autogestión del paciente pluripatológico con insuficiencia cardíaca: un ensayo controlado aleatorizado.		

1. Calidad c-tecnica

Calidad científica y viabilidad técnica y económica de la propuesta. Máximo 50 puntos.

El proyecto deberá contar con un mínimo de 30 puntos en este apartado para poder resultar financiado.

Resulta difícil valorar la calidad científica y viabilidad técnica de la propuesta, porque se presenta un proyecto de investigación mucho más amplio que el que realmente se pretende que sea financiado. Esta es la principal limitación que presenta el proyecto. De hecho, aunque el proyecto presentado tiene clara relevancia científico-técnica, supone innovación, por medio de desarrollo de una herramienta mHealth que impactará en la calidad de los servicios de salud, y puede contribuir en la consolidación de la línea de investigación que siguen las investigadoras principales (IP) del proyecto y su red de colaboradores expertos, una parte significativa del proyecto descansa en la propuesta de un ensayo clínico aleatorizado, multicéntrico, enmascarado para la evaluación con dos grupos: un grupo control (práctica clínica habitual) y un grupo de intervención (práctica clínica habitual junto con intervención de mHealth diseñada ad hoc). Tal y como señalan los investigadores, la ejecución de este proyecto tendría una duración de 4 años, por lo que limitan su solicitud a la primera parte del mismo, es decir, al diseño y validación de contenido de la herramienta de mHealth. No cabe duda del interés y necesidad de la propuesta, pero en su conjunto, es decir, tal y como aparece especificada en la memoria del proyecto, la parte de la memoria relacionada con el ensayo clínico no se corresponde con el proyecto para el que se solicita financiación y, por otro lado, falta seguridad sobre si el equipo obtendrá finalmente financiación para esa "segunda parte" del proyecto: la realización del ensayo clínico que es realmente la aplicación clínica con los pacientes. Por tanto, la falta de este ensayo clínico en la propuesta actual resta interés a la misma y quizá debería haberse planteado este proyecto en convocatorias de investigación que permitieran la ejecución temporal total del proyecto, incluido el ensayo clínico. No obstante, hay que valorar que el proyecto, desde el punto de vista de la viabilidad de su #primera parte# y, potencialmente de la #segunda parte#, cuenta con un equipo con conocimientos y experiencia previa en proyectos similares, congruentes con el historial previo del equipo, y que la calidad científico-técnica del proyecto aparece reflejada en la buena fundamentación teórica descrita y articulación del proyecto en términos de objetivos, método y plan de trabajo.

Puntuación 0 a 50: 39

2. Equipo Inv.

Equipo de investigación. CV del IP o IPs.

Valoración de la composición y experiencia del equipo para llevar a cabo el proyecto: Tendrá una puntuación máxima de 20 puntos y deberá tener un mínimo de 10 puntos en este criterio para ser financiado.

Tanto la composición como la experiencia del equipo para llevar a cabo el proyecto pueden considerarse altamente apropiadas. Respecto a las dos investigadoras principales, destacar su amplia trayectoria investigadora, de más de 15 años, y especialmente su relevancia en la transferencia del conocimiento. Aunque el número de publicaciones en revistas de impacto no es especialmente elevado, destaca su actividad en proyectos de investigación directamente relacionados con el ámbito de solicitud del proyecto, señalando igualmente su pertenencia a diferentes redes que abordan la alfabetización en salud en diferentes contextos y problemáticas de la salud pública. No cabe duda que las líneas de investigación previas de las IP son las

idóneas para llevar a cabo el proyecto que se presenta, orientadas a la psicología de la salud, la enfermería (temática de las revistas empleadas para la difusión de los resultados de investigación), la gestión de la diversidad en salud y la alfabetización en salud. Entre sus intereses también se encuentra la aplicación de estrategias de ludificación (gamificación) para mejorar la formación e implicación de los pacientes en la adherencia terapéutica, lo que resulta de interés para el presente proyecto. Destacar la existencia de patentes, registros en la propiedad intelectual y los premios otorgados por las herramientas diseñadas. Las IP tienen, por lo tanto, un perfil investigador adecuado con reconocida experiencia, señalando como principal limitación el número no excesivamente significativo de publicaciones en revistas de impacto. Otro punto fuerte del equipo investigador lo constituye sin duda el equipo multidisciplinar (psicólogos/as, enfermeras/os, facultativos especialistas de área, ingenieros/as informático, fisioterapeutas y licenciados en ciencias de la actividad física y del deporte) y multicentro que lo constituye. Es de destacar la participación de miembros de la Universidad de Cádiz y del Servicio Andaluz de Salud, lo que constituye un claro recurso a favor en la transferencia del conocimiento. El equipo de investigación que conforma el presente proyecto tiene experiencia y capacitación suficiente para llevar a cabo la propuesta y destaca por su experiencia en el desarrollo de seis productos software relacionados con la gestión de la salud con registro de propiedad intelectual.

Puntuación 0 a 20: 18

3. Impacto result.

Impacto y resultados esperados, publicaciones en Q1, patentes, etc. Máximo 10 puntos.

Se requiere un mínimo de 3 puntos en este apartado para poder resultar financiado.

De acuerdo con la descripción en la memoria de investigación, se espera que los resultados de este proyecto contribuyan a mejorar el nivel de alfabetización en salud, la autogestión y la adhesión terapéutica de los pacientes y, por tanto, contribuyan a reducir en el número de ingresos hospitalarios al año por descompensación y las tasas de incidencia de mortalidad. En conjunto, pues, un impacto notable en la mejora de la atención y gestión sanitaria. Sin embargo, ese potencial impacto queda mermado en parte por las razones señaladas en el apartado 1, en concreto, por no incluirse en este proyecto concreto la realización del ensayo clínico aleatorizado con un total de 236 pacientes (distribuidos en grupo experimental y grupo control), ya que sin dicho ensayo no puede valorarse el impacto planteado por los investigadores. El equipo se propone a publicar los resultados del proyecto en Open Access en revistas científicas internacionales de impacto y Q1 y con la expectativa de que los resultados tengan una amplia difusión en congresos internacionales. Sin embargo, de nuevo, esa potencial difusión en revistas y congresos quedaría limitada en parte por los problemas señalados en el apartado 1, ya que sin la realización del ensayo clínico no es posible disponer de resultados de salud tras la aplicación de la herramienta y, por tanto, también el potencial de difusión de los resultados del proyecto quedaría limitado. Finalmente, habría que destacar que la herramienta de mHealth que desarrollará el proyecto tiene un potencial muy alto de patentabilidad y este es un resultado esperado destacable.

Puntuación 0 a 10: 7

Puntuación final

Puntuación final.

Puntuación 0 a 80: 64,00
